# CT imaging of thyroid cartilage invasion in glottic laryngeal cancer: correlation of traditional semiotics and texture analysis with histopathology

**DOI:** 10.1007/s11547-026-02262-5

**Published:** 2026-07-15

**Authors:** Leonardo Calamandrei, Linda Calistri, Eleonora Bicci, Alberto Zasa, Luca Mazzetti, Sebastiano Paolucci, Francesco Mungai, Luigi Bonasera, Oreste Gallo, Vittorio Miele, Cosimo Nardi

**Affiliations:** 1 Department of Radiology, Santa Maria Annunziata Hospital, Via Antella 58, 50012 Florence, Italy; 2 Department of Experimental and Clinical Biomedical Sciences, University of Florence, Largo Brambilla 3, 50134 Florence, Italy; 3 Department of Radiology, Emergency Radiology Unit, Careggi University Hospital, Largo Brambilla 3, 50134 Florence, Italy; 4 Department of Otorhinolaryngology, Careggi University Hospital, Largo Brambilla 3, 50134 Florence, Italy; 5 Department of Health Physics, Careggi University Hospital, Largo Brambilla 3, 50134 Florence, Italy; 6 Department of Experimental and Clinical Medicine, Otolaryngology - Head and Neck Surgery Unit, University of Florence, Largo Brambilla 3, 50134 Florence, Italy

**Keywords:** Radiomics, Texture analysis, Computed tomography, Glottic squamous cell carcinoma, Laryngeal cancer, Thyroid cartilage invasion

## Abstract

**Objectives:**

To evaluate the diagnostic performance of contrast-enhanced CT and CT-based radiomics in detecting thyroid cartilage invasion in patients with laryngeal squamous cell carcinoma (LSCC), using histopathology as the reference standard.

**Method:**

This retrospective single center study included 60 patients with histologically confirmed glottic LSCC who underwent pre-treatment contrast-enhanced CT between January 2015 and December 2025. Two head and neck radiologists independently assessed thyroid cartilage invasion based on established imaging criteria. Texture analysis was performed on manually segmented thyroid cartilage volumes, extracting 107 textural features. Feature selection and model construction were performed using LASSO logistic regression, whereas diagnostic performance was evaluated using receiver operating characteristic (ROC) curve.

**Results:**

CT-based radiologist assessment showed moderate diagnostic performance in detecting thyroid cartilage invasion, with 63.3% sensitivity, 90.0% specificity and 76.7% accuracy (Cohen’s κ = 0.534). Radiomics analysis identified shape-related and textural features as potential markers of cartilage invasion. The final radiomics model, incorporating “Shape_Maximum 2D Diameter Slice” and “glcm_Imc1,” achieved an AUC of 0.73, 81% sensitivity, 55% specificity and 67% accuracy. While radiologist evaluation offered higher specificity, radiomics demonstrated greater sensitivity, particularly in detecting subtle early-stage infiltration.

**Conclusions:**

Radiologist assessment and radiomics demonstrated different diagnostic strengths in the evaluation of thyroid cartilage invasion in glottic cancer. Although radiomics alone was not sufficient to guide clinical decision-making, the present findings suggested that it may provide additional quantitative information beyond visual assessment. Further studies will be needed to determine whether integration of radiomic features into the diagnostic workflow can improve the assessment of thyroid cartilage invasion.

## Introduction

Laryngeal cancer is a malignant neoplasm predominantly arising from the squamous epithelium of the larynx [1, 2]. It is the second most common malignancy of the head and neck area, with squamous cell carcinoma representing its most common histotype [1–5]. Tobacco smoking and alcohol consumption remain the most significant risk factors, while human papillomavirus infection and exposure to occupational carcinogens are implicated as contributory factors [6–10]. Despite the remarkable progress in our understanding of its pathophysiology and oncogenesis processes, overall mortality of laryngeal cancer remains substantial, largely due to late-stage diagnosis and the aggressive nature of advanced disease [10–14].

Therapeutic management of laryngeal cancer involves a range of surgical and non-surgical approaches [6, 10–12]. Localized lesions often benefit from organ-preserving methods including accelerated radiotherapy or larynx-sparing surgery, whereas tumors in advanced stages particularly those exhibiting extensive cartilage invasion ‒ typically require aggressive surgical interventions such as total laryngectomy [6, 13–16]. In these cases, decision-making is not only influenced by tumor size, but also by pre-treatment laryngeal function, with surgery often preferred when organ function is significantly compromised [1, 2, 6–8, 12].

Accurate staging is critical in the management of glottic carcinoma and is primarily based on the TNM classification, wherein cartilage invasion serves as a pivotal criterion [2, 17]. Involvement of the thyroid or cricoid cartilage is considered indicative of advanced stages (T3–T4), which are associated with a less favorable prognosis and generally necessitate more radical treatment techniques [1, 3, 7, 11, 12, 18]. Thus, determining the extent of cartilage infiltration is essential for both prognostic evaluation and treatment planning [11, 12].

Computed tomography (CT) imaging plays a central role in the diagnostic and prognostic processes, especially regarding the assessment of laryngeal cartilage invasion [18, 19]. CT is mainly valuable in emergency settings and preoperative evaluation due to its rapid acquisition time and effectiveness in depicting bone-cartilage structures [17, 20]. Although magnetic resonance imaging provides better soft tissue characterization than CT, it is impeded by overall lower availability and longer acquisition times with consequent high susceptibility to motion artifacts in such fine and mobile structures as the glottis [17, 21, 22]. For these reasons, CT remains a mainstay in clinical practice because it efficiently delineates critical anatomical details necessary for assessing airway compromise and structural involvement [17, 23–25].

In the context of diagnostic and prognostic assessment, emerging techniques such as texture analysis have been revolutionizing radiological evaluation in the last few years by quantifying textural heterogeneity within tumor tissue [26–29]. This approach leverages advanced image processing algorithms to detect subtle differences in tissue composition, thereby enhancing the predictive accuracy for treatment responses and overall prognoses [24, 30, 31]. Texture analysis is increasingly being integrated into the diagnostic workflow, providing complementary information that reinforces the role of CT in the comprehensive management of laryngeal cancer [24, 31–33].

The objective of this study was to evaluate the efficacy of both conventional CT semiotics and more modern texture analysis techniques in the detection of thyroid cartilage infiltration by glottic cancer, by comparing them with histology.

## Materials and methods

### Patients’ selection

A single-center, observational and retrospective study was carried out on patients with squamous cell carcinoma of the larynx (LSCC) that underwent CT at staging between January 2015 and December 2025.

Inclusion criteria were:

- Over 18 years of age;
- Histological diagnosis of LSCC;
- Tumor involving the glottis;
- Pre-treatment unenhanced and contrast-enhanced CT.

Exclusion criteria were:

- No histological evaluation of thyroid cartilage involvement;
- Previous exposure to radiation therapy in head and neck area;
- Previous surgery in head and neck area;
- CT examination performed in different centers;
- Tumor confined to supraglottis or subglottis, without glottis involvement.

**Fig. 1:**
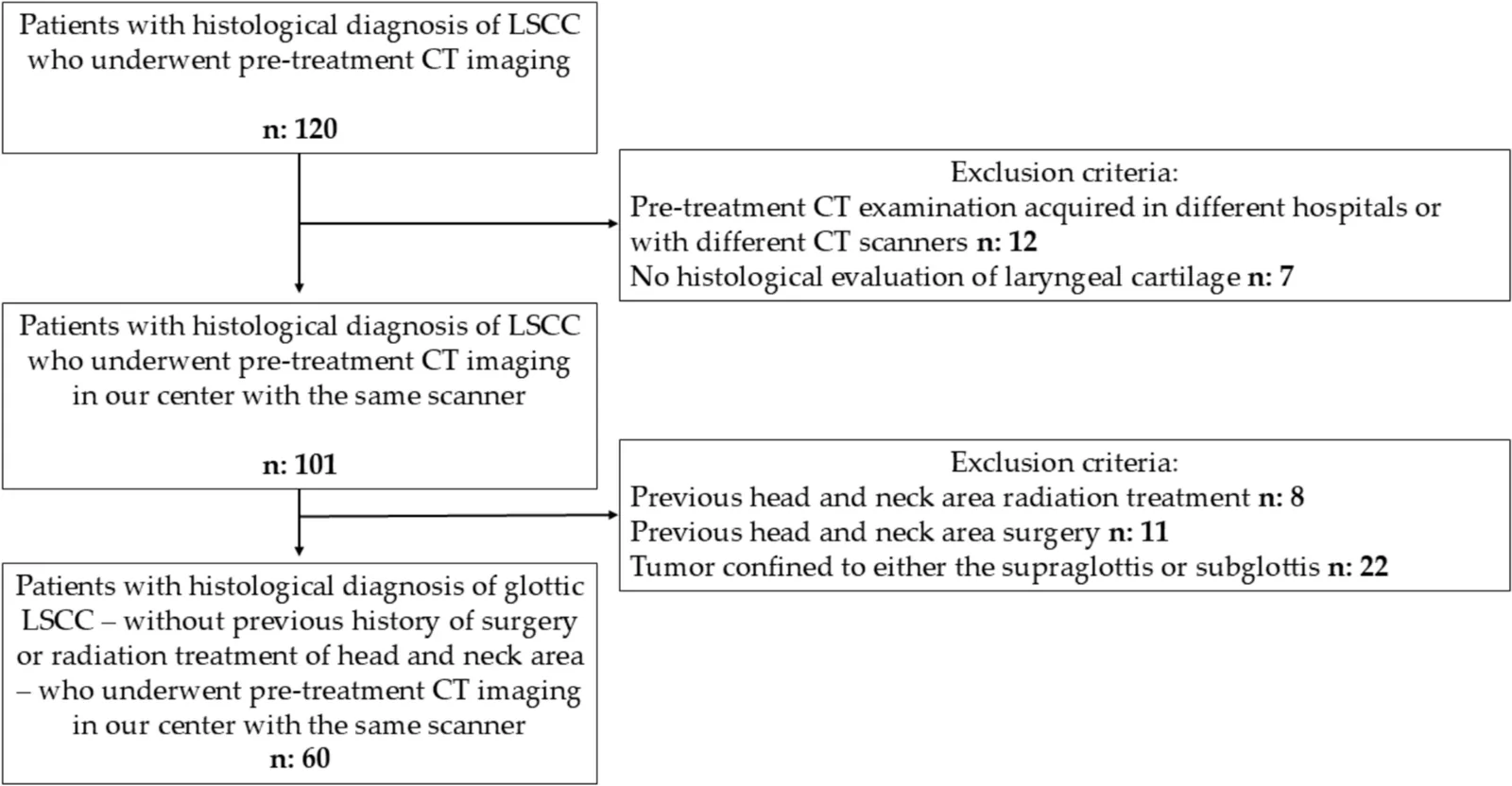
Workflow of patients’ selection following inclusion and exclusion criteria. LSCC: laryngeal squamous cell carcinoma

Patient selection algorithm was shown in Fig. 1. Starting from a population of 120 patients, 12 of them were excluded because pre-treatment baseline CT was acquired in different hospitals, while 7 patients did not have histological documentation of cartilage status. Of the remaining 101 patients, 8 of them underwent previous radiation therapy, 11 patients had previous surgery and in 22 cases the tumor was confined either to the supraglottis or subglottis without involvement of the glottis. The final sample included 60 patients (13 women and 47 men; mean age 72 years, median age 73 years, range 50–91 years) with first diagnosis of glottic LSCC, baseline contrast-enhanced CT examination and histological report documenting thyroid cartilage status.

### CT acquisition protocol and cartilage assessment

All CT examinations were conducted using a 128-slice helical CT scanner (SOMATOM Definition Flash, Siemens Healthcare, Erlangen, Germany), with imaging parameters optimized for high-resolution acquisition: tube voltage 100 kV, pixel resolution 0.465 mm, field of view set to 200 mm, 1-mm slice thickness with a corresponding 1-mm reconstruction interval, tube current time product 186 mAs, gantry rotation time 0.5 s, beam collimation 128 × 0.6 mm, pitch 0.8, and application of a 131 s/3 reconstruction kernel. Each patient underwent both non-contrast and contrast-enhanced scans. The latter was acquired 60 s after intravenous administration of iopromide (Ultravist 370, Bayer Healthcare) at a dosage of 1.5 mL/kg and infused via power injector at 3 mL/s, followed by a 40 mL saline flush. The scan range extended from the skull base to the thoracic inlet. Post-processing involved reconstructing the acquired data into 1-mm axial slices and generating multiplanar reformats in sagittal and coronal orientations, both aligned with the glottic plane to ensure anatomical consistency.

All CT examinations were independently reviewed by two radiologists with 13 and 5 years of experience in head and neck imaging, respectively. Each CT scan was assessed for thyroid cartilage invasion according to the criteria proposed by Becker et al. [34] and staged consequently. Such assessment was performed in a blinded fashion, with radiologists unaware of the post-surgical pTNM stage and histological cartilage status.

**Fig. 2:**
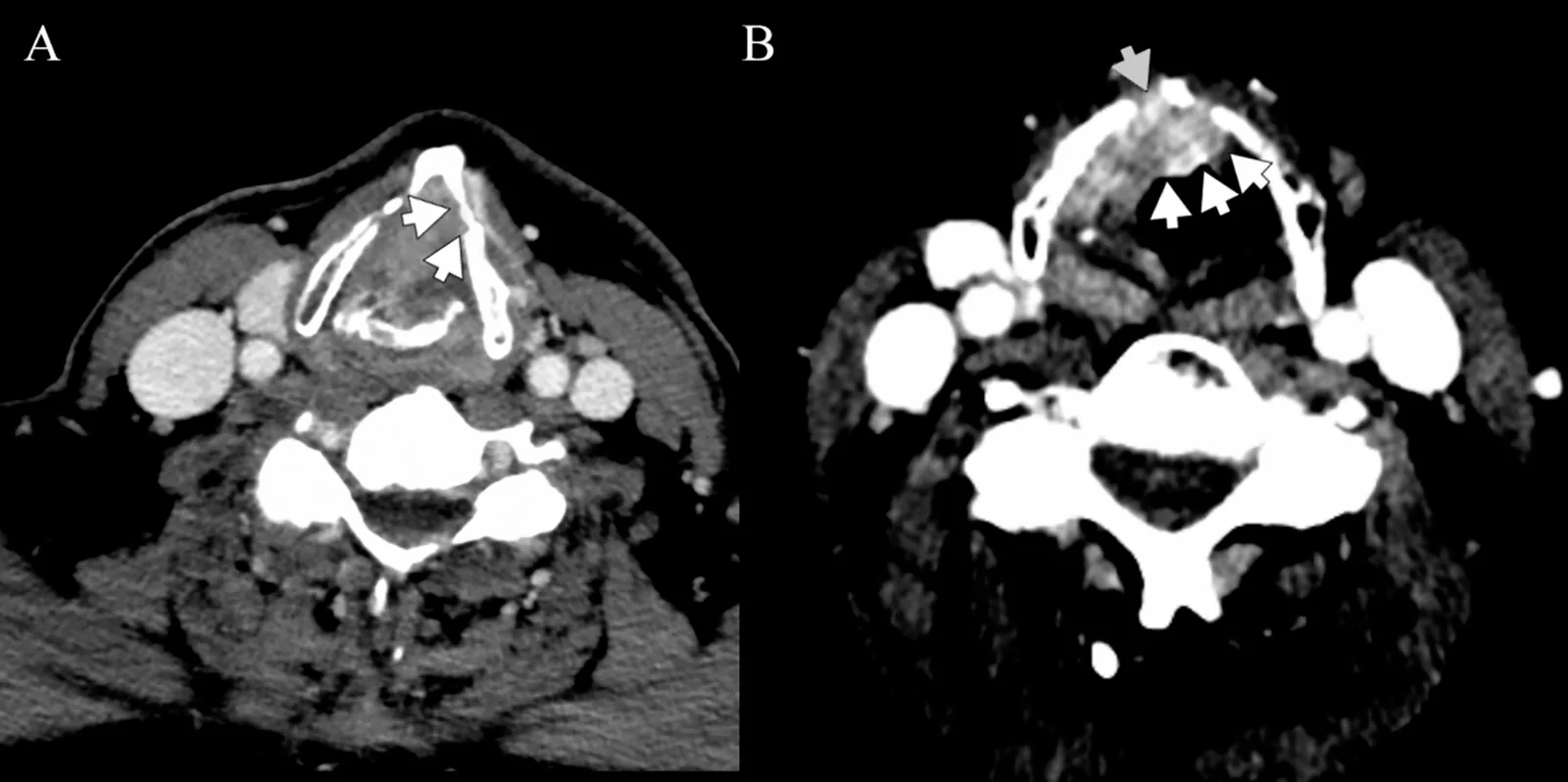
Axial contrast-enhanced CT images demonstrating two different trans glottic laryngeal tumors. A. Cortical irregularity and focal loss of the inner margin of the thyroid cartilage adjacent to the tumor (white arrows), indicating early cartilage invasion (inner cortical erosion). B. Nodular soft-tissue enhancement extending into the thyroid cartilage (white arrows) with discontinuity of the outer cortical boundary (gray arrow), consistent with complete cartilage involvement (full-thickness infiltration)

Inner cortical erosion was defined as any focal thyroid cartilage defect in direct continuity with the tumor mass, whereas full-thickness invasion was characterized by tumor tissue replacing the thyroid cartilage or extending into extralaryngeal soft tissues on contrast-enhanced images (Fig. 2). Thyroid cartilage sclerosis was excluded from analysis because of its low specificity (40%) for detecting tumor invasion [17, 34, 35].

For analytical purposes, the presence of either inner cortical erosion or full-thickness invasion was classified as a positive radiological finding, whereas the absence of both was classified as negative. Radiological findings were then correlated with histopathological evidence of thyroid cartilage invasion. Finally, diagnostic performance metrics including sensitivity, specificity, positive predictive value (PPV), negative predictive value (NPV), and overall accuracy were calculated.

### Texture analysis

**Fig. 3:**
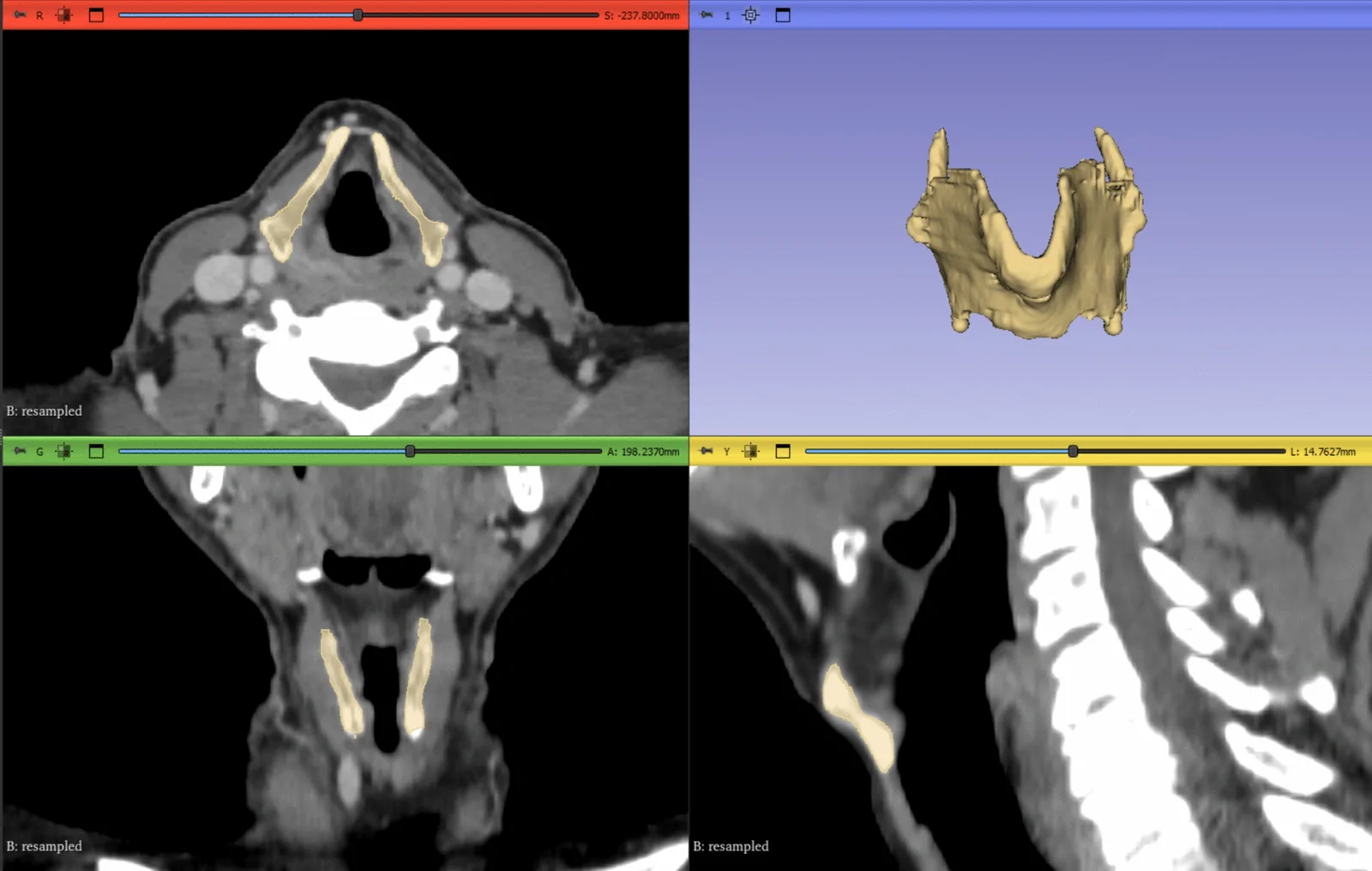
Segmentation with 3D render of thyroid cartilage on 3D slicer v.5.8.1

Contrast-enhanced CT scans were imported into the open-source software 3D Slicer (version 5.8.1; https://www.slicer.org/) and resampled using the “Resample Scalar Volume” module to standardize voxel sizes across all scans (1 × 1 × 1 mm in the x, y, and z axes respectively). Subsequently, the entire volume of thyroid cartilages was manually segmented on a slice-by-slice basis by a third radiologist with over 5 years of experience in head and neck imaging. Radiomic feature extraction was performed using the SlicerRadiomics extension within 3D Slicer. Segmentation on 3D slicer was shown in Fig. 3. A total of 107 features were extracted, encompassing categories including First-Order Statistics, 3D Shape-Based Features, Gray Level Co-occurrence Matrix (GLCM), Gray Level Size Zone Matrix (GLSZM), Gray Level Run Length Matrix (GLRLM), Neighboring Gray Tone Difference Matrix (NGTDM), and Gray Level Dependence Matrix (GLDM). All features were derived in accordance with the PyRadiomics framework, an open-source Python package designed for radiomic analysis of medical imaging data (https://pyradiomics.readthedocs.io).

### Statistical analysis and radiomic modeling

A total of 60 patients were divided into two groups: 30 patients with histologically proven thyroid cartilage invasion (group 1) and 30 patients without cartilage invasion (group 0).

A total of 107 radiomic features were extracted for each patient. To identify features showing significant differences between the two groups, the following procedure was applied:

1. Normality and homogeneity of variance tests. For each feature, normality within each group was assessed using the Shapiro–Wilk test, and equality of variances between groups was assessed using the Levene test.
2. Group comparisons. Features satisfying normality and homoscedasticity assumptions (*p* > 0.05 for both Shapiro–Wilk and Levene tests) were compared using Student’s t-test. If at least one assumption was violated (*p* < 0.05), the Mann–Whitney U test was used.
3. Statistical significance. Features with *p* < 0.05 were considered statistically significant.
4. Direction of difference. For significant features, the group with the higher mean (if t-test was used) or median (if Mann–Whitney U test was used) was recorded.

Radiomic models were constructed for each analysis based on the identification of statistically significant features. Logistic regression with LASSO (Least Absolute Shrinkage and Selection Operator) or ridge regression penalization was used to build the models. LASSO was applied either to all 107 features or to subsets of significant features to perform feature selection and regularization. Ridge regression was applied to manually selected feature subsets. The regularization parameter λ was optimized using fivefold cross-validation to maximize the area under the receiver operating characteristic curve (AUC). Weighted logistic regression was applied in cases of class imbalance (≥ 70% of patients in one group).

Model performance was assessed using AUC and the optimal classification threshold was determined using the Youden index. Confusion matrices and performance metrics, including accuracy, sensitivity, specificity, F1-score, and Matthews correlation coefficient were computed accordingly.

Additionally, heatmaps and dendrograms were generated to assess correlations among the 107 features across patients. Spearman correlation coefficients were calculated for each patient pair and hierarchical clustering was performed using Ward’s method.

## Results

### Imaging assessment

Interobserver agreement for the assessment of thyroid cartilage invasion was substantial, with a concordance rate of 72.0%. Twenty-two out of the 60 patients investigated (36.7%) were classified as radiologically positive for cartilage involvement, whereas the remaining 38 patients (63.3%) were classified as negative. Histopathological analysis confirmed thyroid cartilage invasion in 19 out of the 22 radiologically positive patients, yielding 3 false-positive cases (13.0%). Among the 38 radiologically negative patients, 11 patients were found to have cartilage invasion on histology, representing false-negative cases (28.9%). Based on these results, CT evaluation demonstrated sensitivity of 63.3%, specificity of 90.0%, positive predictive value (PPV) of 86.4%, negative predictive value (NPV) of 71.1%, and overall accuracy of 76.7%. Agreement between radiological and histopathological assessments was moderate (Cohen’s kappa 0.534). The distribution of radiological findings and their histopathological correlation was summarized in Table 1.

**Table 1:** Performance of radiologist assessment for the detection of thyroid cartilage invasion on contrast-enhanced computed tomography (CT)

CT	Histology		
	Positive	Negative	Total
Positive	19	3	22
Negative	11	27	38
Total	30	30	60

### Texture analysis and radiomics modeling

#### Feature distribution

Among all extracted features, three shape-related features—Major Axis Length, Maximum 2D Diameter (Slice), and Surface-to-Volume Ratio—showed significant differences between the 2 patient groups (group 0: no cartilage involvement on histology; group 1: confirmed cartilage involvement on histology) at unadjusted *P* values.

The descriptive statistics and corresponding *p* values are summarized in Table 2.

**Table 2:** Descriptive statistics for the three statistically significant features between the two groups

Significative features	*p* value	Mean group 0	Mean group 1
shape_MajorAxisLength	0.045	61.38	64.94
shape_Maximum2DDiameterSlice	0.008	49.63	53.31
shape_SurfaceVolumeRatio	0.049	0.806	0.731

Group 0 and group 1 represented patients without and with histologically proven thyroid cartilage invasion, respectively. Statistical significance was determined using T-test

#### Model selection

Two features were retained as most predictive for group classification using LASSO regression:

- Shape_Maximum 2D Diameter Slice
- glcm_Imc1

**Fig. 4:**
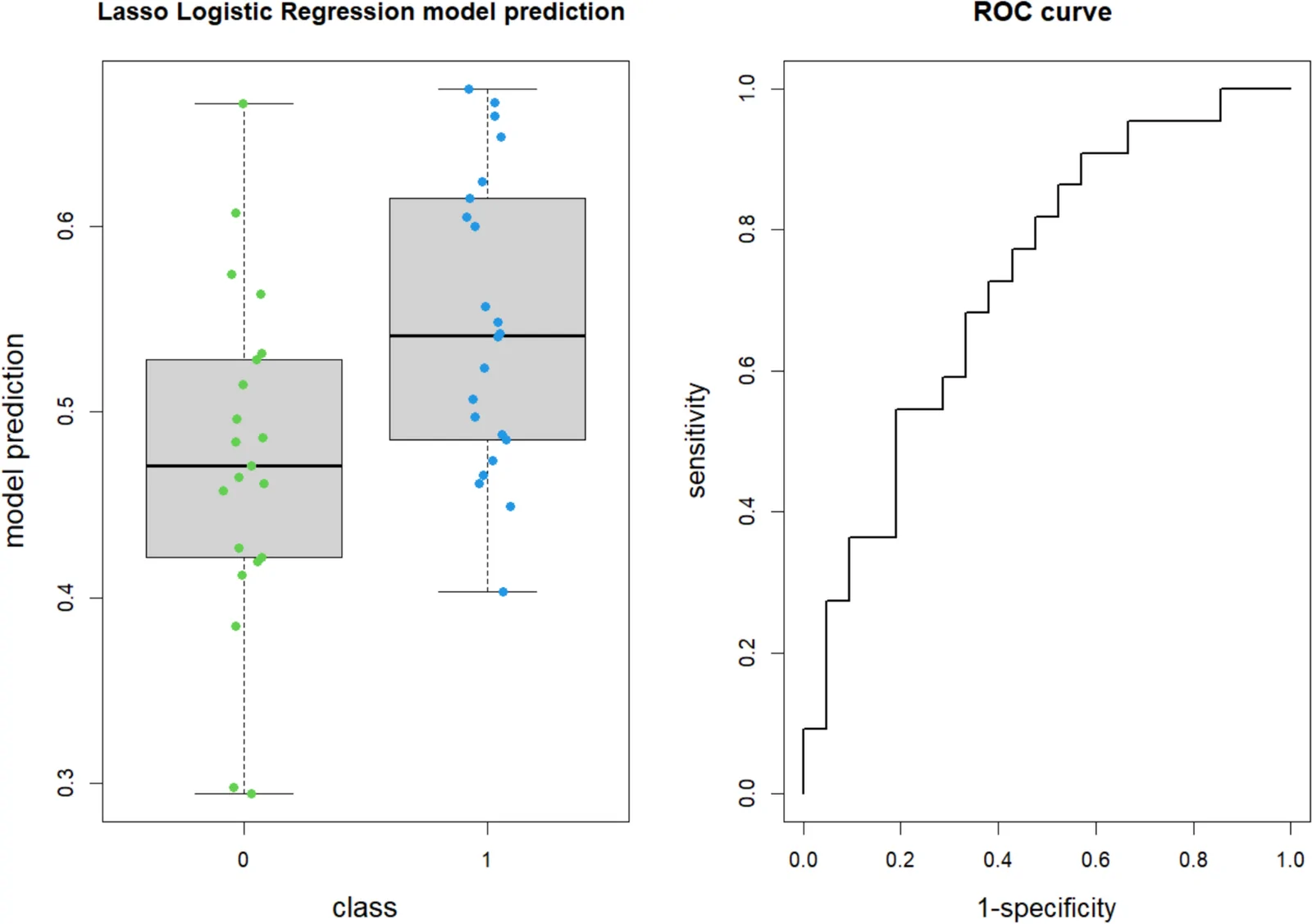
Boxplots and ROC curve for the radiomics model predicting thyroid cartilage invasion in glottic laryngeal cancer. Boxplots showed the distribution of selected radiomic features (Shape Maximum 2D Diameter Slice and glcm_Imc1) in patients without (0) and with (1) thyroid cartilage invasion

A radiomic model built on these features achieved AUC of 0.73 (95% CI 0.58–0.88; DeLong test). Boxplots and ROC Curve were shown in Fig. 4.

At the optimal cut-off determined by the Youden index (threshold = 0.54), the performance metrics were as follows: sensitivity 0.81, specificity 0.55, PPV 0.63, NPV 0.75, ACC 0.67, F1-score 0.74, Balanced Accuracy 0.68, and Matthews Correlation Coefficient 0.37. The corresponding confusion matrix at 0.54 threshold was shown in Table 3.

**Table 3:** Confusion matrix for the radiomics model predicting thyroid cartilage invasion

Radiomic model	Histology	
	Actually negative	Actually positive
Predicted negative	TN: 16	FN: 6
Predicted positive	FP: 14	TP: 24

The rows represented the true classes as indicated by histology and the columns indicated the classes as predicted by the model. TP: true positives. TN: true negatives. FP: false positives. FN: false negatives. Classification was based on the Youden-index threshold (0.54) applied to model predictions

## Discussion

The present study investigated the ability of CT-based radiomic features to detect thyroid cartilage invasion in patients with glottic cancer and compared their performance with the visual assessment by two head and neck radiologists.

### Visual assessment

CT evaluation proved a moderate diagnostic performance in the detection of thyroid cartilage invasion, with sensitivity of 63.3%, specificity of 90.0%, PPV of 86.4%, NPV of 71.1%, and overall accuracy of 76.7%, demonstrating moderate agreement with histopathological findings (κ = 0.534). These results were consistent with previously published data, although values for CT performance widely varied across studies, in fact sensitivity ranging from 40% to 80% and specificity from 70% to 95% for the detection of thyroid cartilage invasion on CT imaging [35–38]. Pietragalla et al. [35] stated that CT findings of inner cortex erosion of the thyroid cartilage were highly predictive of true histological infiltration, especially when associated with extension beyond the cartilage framework. However, they also observed that the absence of CT-detectable erosion did not fully exclude microscopic cartilage involvement. In line with these observations our data confirmed that CT was highly specific for detecting overt cartilage erosion, but such imaging technique lacked adequate sensitivity as minimal or focal invasions were often overlooked [2, 23]. Locatello et al. [38] also highlighted this limitation, noting that distinguishing between inner cortex (cT3) and full-thickness invasion (cT4a) remained challenging even with contemporary CT protocols. In our interpretation, the low sensitivity observed in the current series and consequently the high false-negative cases (28.9%) reflected this same diagnostic challenge. Therefore, while CT remains to this day the first-line technique for preoperative assessment of thyroid cartilage infiltration, subtle cortical breaches often go undetected, underscoring the need for close clinicopathological correlation for accurate staging.

### Interpretation of the significant shape features and relation to imaging signs

Univariate analysis identified three shape-related radiomic features that differed between patients with confirmed thyroid cartilage invasion and patients without evidence of invasion at unadjusted significance thresholds: Shape_Major_Axis Length, Shape_Maximum_2D Diameter Slice, and Shape_Surface_Volume Ratio.

- *Shape_Major_Axis Length* In three-dimensional radiomic analysis, “Major Axis Length” describes the maximum linear extent of the ellipsoid that best fits the lesion volume [39]. It is a morphological metric indicative of the tumor size/elongation [27, 30, 40–43] and a global measure of extent/elongation in three dimensions [39, 44]. In our cohort, values were lower in patients without invasion and relatively larger (5.8% higher mean value in group 1) in patients with confirmed cartilage involvement. In a previous study by Oki et al. [44] this shape feature was found to correlate with the rate of cervical lymph node metastases in patients with oral cavity carcinoma. It was therefore hypothesized by the authors that since larger tumors were more prone to regional dissemination [8, 9, 44, 45], it would be reasonable for Major Axis Length ‒ a geometric feature linked to tumor volume ‒ to predict the presence of neck metastases. Similarly, in our study, a high mean value of Major Axis Length in positive patients could be consistent with an increase in the maximal geometric extent of the segmented structures when tumor has produced deformation or replacement of cartilage tissue.
- *Shape_Maximum 2D Diameter Slice* measures the largest in-slice Euclidean distance across the object (the maximum cross-sectional diameter measured on any axial slice) [27, 40, 41, 43, 46]. In the same way as Major Axis Length, Shape_Maximum 2D Diameter Slice is a size/extent metric but constrained to a single plane [39]. Therefore, Maximum 2D Diameter Slice is particularly sensitive to focal expansions, bulging, or loss of cortical contour visible on axial images [24, 26, 44, 47]. In our cohort, larger values of this feature were found in patients with confirmed cartilage invasion (7.4% higher mean value in group 1 vs group 0). Much like shape_Major axis length, this increase in maximal cross-sectional size could indicate an association with invasion or adjacent tumor bulk, given the similar conceptual nature of the two shape features [39].
- *Shape_Surface_Volume Ratio* is the ratio between the surface area and the volume of the segmented object [26, 40, 47, 48]. Higher values correspond to structures with relatively large surface area for a given volume (i.e., more “surface complexity” or thin/irregular shapes), whereas lower values indicate relatively greater volume with less surface per unit volume (i.e., more compact/bulky shapes) [49, 50]. To the best of the authors’ knowledge, this feature has not been identified as a significant predictor in head and neck cancer itself, either at the time of staging or during follow-up. However, it was previously reported as a predictor of aggressiveness in other malignancies, including breast cancer, and bladder cancer [49, 50]. In our data Surface Volume Ratio was higher in non-invaded cartilage and lower in invaded cartilage (9.3% lower value in group 1 vs. group 0). This could be an expression of the concept that cartilage invaded by tumor becomes relatively bulkier (volume increases disproportionately to surface area) or that the segmentation includes more bulk due to cortical cartilage replacement.

The two size/extent metrics (Major Axis Length and Maximum 2D Diameter Slice) and the surface/volume index provide complementary information [39, 43]. Size metrics capture macroscopic enlargement or deformation, whereas the surface/volume ratio captures a change in shape compactness versus surface complexity [39]. These quantitative changes could also be consistent with the two traditionally identified imaging signs used by radiologists to examine thyroid cartilage invasion ‒ inner cortex erosion and full-thickness infiltration [34]—which were also assessed in the current study. Inner cortex erosion, usually defined as a subtle focal cortical thinning or irregularity [2, 35], is a relatively early sign [34, 35, 51] and as such may primarily alter local surface complexity and borderline irregularities. Such changes might produce small decreases in surface regularity and subtle shifts in the surface/volume relationship, although these changes were difficult to detect with purely global size metrics such as the above-mentioned features. Full-thickness infiltration consists of macroscopic deformation or replacement of cartilage by tumor tissue [17, 42] and as such could both increase maximal diameters and the major axis length of the invaded cartilage with a tendency to decrease the surface/volume ratio since the anatomical structure becomes bulkier and volumetrically dominated by infiltrative tissue.

Therefore, Major Axis Length, Maximum 2D Diameter Slice and Surface Volume Ratio could quantify in numeric form the same geometric phenomena that radiologists qualitatively identified as an expression of thyroid cartilage invasion. In this sense radiomics can be read as a method to objectivize and quantify the radiologist’s interpretation by visual assessment and as an attempt to reduce reliance on subjective impression and to enable reproducible measurement.

### Radiomics model

LASSO feature selection retained Maximum 2D Diameter Slice together with glcm_Imc1 as most significant features to produce the final predictive model. While the possible meaning and interpretation of Maximum 2D Diameter Slice in regards to thyroid cartilage infiltration was described in the previous paragraph, glcm_Imc1 is a second-order texture descriptor derived from the gray-level co-occurrence matrix [30, 39, 43, 50]. This feature encodes information about the dependency/correlation between neighboring gray levels and it is sensitive to the degree of textural complexity and spatial relationships of intensities within the segmented region [26, 27, 39, 41, 47]. Glcm_Imc1 has been previously shown to be a measure of textural heterogeneity in tumoral tissue [24, 31, 47]. In head and neck cancer radiomics, Safakish et al. [52] stated that this feature was associated with treatment outcomes when incorporated into a multi-feature predictive model.

Changes in glcm_Imc1 in invaded cartilage could be interpreted as variations in tissue composition at the cartilage–tumor interface resulting in higher texture heterogeneity, such as changes in enhancement, partial volume effect and mixed cartilage and tumor attenuation that might otherwise not be captured by pure shape metrics. Patients with positive histology for cartilage infiltration showed overall lower values of glcm_Imc1, possibly indicating the replacement of the normally organized cartilage texture with more heterogenous tumor tissue or a loss of normal cartilage textural patterns. The radiomics model achieved an AUC of 0.73 (95% CI 0.58–0.88), with sensitivity of 0.81, specificity of 0.55, PPV of 0.63, NPV of 0.75, and accuracy of 0.67. These results indicated moderate discriminative power, with the model performing better at excluding invasion than confirming its presence. In contrast, the visual assessment by radiologists proved higher specificity (90.0%) and overall accuracy (76.7%) but lower sensitivity (63.3%) than the radiomics model. Therefore, in the current study radiologists achieved reliable confirmation of the absence of thyroid cartilage invasion, but exhibited moderate ability to identify subtle or early infiltration. These findings suggested that while radiomics can capture structural and textural alterations in the laryngeal cartilage, its current performance did not surpass that of expert radiologists. In summary, radiomics demonstrated higher sensitivity in detecting subtle structural and textural alterations, whereas radiologists achieved greater specificity reducing the risk of false positives. As such, radiomics may currently be best regarded as a complementary tool to the visual radiological assessment rather than serving as a standalone diagnostic method, thereby enhancing the detection of subtle early changes in thyroid cartilage that might otherwise be overlooked. An additional area of interest would be the development of integrated models combining radiomic features with expert radiological assessment. Such an approach could potentially leverage the higher sensitivity of radiomics together with the high specificity achieved by experienced radiologists, thereby improving overall diagnostic performance. However, the present study was not designed to develop a combined classifier. Given the limited sample size, incorporation of both radiological and radiomic variables into a single predictive model would have increased the risk of overfitting and reduced the robustness of performance estimates. Multicenter studies with larger patient cohorts should explore the feasibility and clinical value of integrated radiologist–radiomics models.

### Limitations and future proposal

Although the study included a balanced cohort of 60 patients, with equal representation of positive and negative cases, the overall sample size was modest. This aspect limited the statistical power and the precision of performance estimates, reflecting a common challenge in head and neck oncology research due to the comparatively low incidence of glottic tumors [1, 3, 36].

Another limitation concerned the segmentation workflow adopted for radiomic analysis. All segmentations were performed by a single dedicated head and neck radiologist in order to ensure methodological consistency across the entire dataset and to minimize variability related to different segmentation strategies. However, no formal interobserver or intraobserver reproducibility analysis was performed. Consequently, the robustness of the extracted radiomic features to segmentation variability was not specifically assessed. Future studies should include repeated segmentations by multiple observers and evaluate feature reproducibility to further strengthen the reliability and generalizability of radiomic models in thyroid cartilage assessment.

In addition to that, all CT examinations were carried out on a single CT scanner with a fixed protocol. While this feature improved internal consistency, it significantly reduced the sample size and at the same time reduced generalizability of our results, as radiomic measures are known to be very sensitive to acquisition and reconstruction parameters [42, 46, 47]. Furthermore, although no independent external validation cohort was available, the study was intentionally designed to prioritize a homogeneous pathological reference standard. Only patients with histopathological confirmation of thyroid cartilage status were included, ensuring direct radiologic–pathologic correlation. While this approach limited the number of eligible patients and precluded external validation, it strengthened the internal validity of the study by reducing uncertainty regarding the reference standard. To address both the limited sample size and generalizability, future studies should involve multicenter cohorts and multiple CT scanners, which would increase patient numbers and ensure broader applicability of radiomics models.

Finally, it could be valuable to apply the same study model to magnetic resonance imaging as well as advanced CT techniques, such as dual-energy CT or photon-counting CT, to explore whether these imaging techniques provide additional structural or textural information capable of further improving the detection of thyroid cartilage invasion.

## Conclusions

The present study emphasized the complementary role of the visual radiological assessment and radiomics in the detection of thyroid cartilage invasion in glottic cancer. When compared to radiomics model, radiologists demonstrated high specificity (90.0%) and adequate overall accuracy (76.7%) but low sensitivity (63.3%). These findings suggested the exclusion with high reliability of thyroid cartilage invasion, though subtle involvement may remain undetected. In contrast, the radiomics model showed high sensitivity (81.0%) and low specificity (55.0%), identifying features that might not be readily apparent on visual assessment, even though an increased risk of false positives must be taken into account. Therefore, although radiomics was not suitable as a standalone diagnostic method in the identification of thyroid cartilage invasion in glottic cancer, it provided complementary information to visual radiological assessment by detecting subtle structural and textural alterations that were not visible in CT images. Future studies should investigate whether the integration of radiomic features with expert radiological assessment can improve diagnostic accuracy, consistency, and clinical decision-making.

## References

[1] Sahafi P, Sadeghi R, Askari E, Sahebkari A, Ghahraman M, Khadivi E et al (2025) Sentinel node biopsy in laryngeal cancer: a systematic review and meta-analysis. Diagnostics. 10.3390/diagnostics15030366 PMC11817158 39941296

[2] Pucetaite M, Farina D, Ryskiene S, Mitraite D, Tarasevicius R, Lukosevicius S et al (2024) The diagnostic value of CEUS in assessing non-ossified thyroid cartilage invasion in patients with laryngeal squamous cell carcinoma. J Clin Med 13(3):891 10.3390/jcm13030891 PMC10856113 38337585

[3] Zheng T, Xiao Y, Yang F, Dai G, Wang F, Chen G (2024) The value of dual-layer spectral detector CT in preoperative T staging of laryngeal and hypopharyngeal squamous cell carcinoma. Eur J Radiol 171:111287 10.1016/j.ejrad.2024.111287 38176085

[4] Consalvo F, De Simone M, Scarpa A, Acerra A, Salzano FA, Fineschi V et al (2024) Challenges and complications in the management of advanced oropharyngeal carcinoma: role of post-mortem diagnosis and future perspectives. J Clin Med. 10.3390/jcm13175198 PMC11396599 39274413

[5] Rahman R, Shaikh MH, Gopinath D, Idris A, Johnson NW (2023) Human papillomavirus and Epstein-Barr virus co-infection in oral and oropharyngeal squamous cell carcinomas: a systematic review and meta-analysis. Mol Oral Microbiol 38(4):259–274 10.1111/omi.12412 37014754

[6] Cho KJ, Kim MS, Cho JH, Park JO, Nam IC, Kim CS et al (2023) Oncological and functional outcomes of partial or total laryngopharyngectomy for hypopharyngeal cancer with thyroid or cricoid cartilage invasion. Acta Otolaryngol 143(5):429–433 10.1080/00016489.2023.2205443 37148316

[7] Geng D, Chen X, Zhao XG, Xu XQ, Su GY, Zhou Y et al (2023) Laryngeal and hypopharyngeal squamous cell carcinoma: association between quantitative parameters derived from dual-energy CT and histopathological prognostic factors. Acta Radiol 64(7):2268–2276 10.1177/02841851221095237 35502811

[8] Chien CY, Wang CP, Lee LY, Lee SR, Ng SH, Kang CJ et al (2023) Indications for elective neck dissection in cT1N0M0 oral cavity cancer according to the AJCC eight edition: a nationwide study. Oral Oncol 140:106366 10.1016/j.oraloncology.2023.106366 36965411

[9] Tran Q, Maddineni S, Arnaud EH, Divi V, Megwalu UC, Topf MC et al (2023) Oral cavity cancer in young, non-smoking, and non-drinking patients: a contemporary review. Crit Rev Oncol Hematol 190:104112 10.1016/j.critrevonc.2023.104112 PMC10530437 37633348

[10] Wellenstein DJ, Woodburn J, Marres HAM, van den Broek GB (2023) Detection of laryngeal carcinoma during endoscopy using artificial intelligence. Head Neck 45(9):2217–2226 10.1002/hed.27441 37377069

[11] Igissin N, Zatonskikh V, Telmanova Z, Tulebaev R, Moore M (2023) Laryngeal cancer: epidemiology, etiology, and prevention: a narrative review. Iran J Public Health 52(11):2248–2259 10.18502/ijph.v52i11.14025 PMC10719707 38106821

[12] Liberale C, Soloperto D, Marchioni A, Monzani D, Sacchetto L (2023) Updates on larynx cancer: risk factors and oncogenesis. Int J Mol Sci. 10.3390/ijms241612913 PMC10454133 37629093

[13] Russo E, Costantino A, Veneroni MV, Festa BM, Pellini R, Campo F et al (2023) Transoral laser microsurgery in recurrent laryngeal cancer: a systematic review and meta-analysis. Laryngoscope 133(6):1425–1433 10.1002/lary.30332 37158265

[14] Campo F, Mazzola F, Bianchi G, Manciocco V, Ralli M, Greco A et al (2023) Partial laryngectomy for naive pT3N0 laryngeal cancer: systematic review on oncological outcomes. Head Neck 45(1):243–250 10.1002/hed.27205 36177857

[15] Broseghini E, Filippini DM, Fabbri L, Leonardi R, Abeshi A, Dal Molin D et al (2023) Diagnostic and prognostic value of microRNAs in patients with laryngeal cancer: a systematic review. Non-coding RNA. 10.3390/ncrna9010009 PMC9966707 36827542

[16] Bertino G, Pedretti F, Mauramati S, Filauro M, Vallin A, Mora F et al (2023) Recurrent laryngeal papillomatosis: multimodal therapeutic strategies. Literature review and multicentre retrospective study. Acta Otorhinolaryngol Ital 43(Suppl. 1):S111–S122 10.14639/0392-100X-suppl.1-43-2023-14 PMC10159644 37698108

[17] Becker M, Monnier Y, de Vito C (2022) MR imaging of laryngeal and hypopharyngeal cancer. Magn Reson Imaging Clin N Am 30(1):53–72 10.1016/j.mric.2021.08.002 34802581

[18] Lechien JR, Maniaci A, Hans S, Iannella G, Fakhry N, Mayo-Yanez M et al (2022) Epidemiological, clinical and oncological outcomes of young patients with laryngeal cancer: a systematic review. Eur Arch Otorhinolaryngol 279(12):5741–5753 10.1007/s00405-022-07466-9 35652941

[19] Zhang H, Zou Y, Tian F, Li W, Ji X, Guo Y et al (2022) Dual-energy CT may predict post-operative recurrence in early-stage glottic laryngeal cancer: a novel nomogram and risk stratification system. Eur Radiol 32(3):1921–1930 10.1007/s00330-021-08265-2 34762148

[20] Stevanovic S, Greguric T, Pazanin L, Ivkic B, Geber A, Hadzavdic A et al (2023) Diagnostic accuracy of preoperative CT and endoscopy staging in early laryngeal cancer. Otolaryngol Head Neck Surg 168(4):769–774 10.1177/01945998221109819 35763371

[21] Mohamad I, Hejleh TA, Qandeel M, Al-Hussaini M, Koro S, Taqash A et al (2023) Concordance between head and neck MRI and histopathology in detecting laryngeal subsite invasion among patients with laryngeal cancer. Cancer Imaging 23(1):99 10.1186/s40644-023-00618-y PMC10585883 37858162

[22] Park CJ, Kim JH, Ahn SS, Lee SK, Koh YW, Kim J (2021) Preoperative MRI evaluation of thyroid cartilage invasion in patients with laryngohypopharyngeal cancer: comparison of contrast-enhanced 2D spin-echo and 3D T1-weighted radial gradient recalled-echo techniques. AJNR Am J Neuroradiol 42(9):1690–1694 10.3174/ajnr.A7213 PMC8423038 34301638

[23] Ravanelli M, Rondi P, Ferrari M, Lancini D, Buffoli B, Borghesi A et al (2024) CT and MR anatomy of the larynx and hypopharynx. Neuroradiology 66(6):883–896 10.1007/s00234-024-03320-3 38418594

[24] Wang W, Liang H, Zhang Z, Xu C, Wei D, Li W et al (2024) Comparing three-dimensional and two-dimensional deep-learning, radiomics, and fusion models for predicting occult lymph node metastasis in laryngeal squamous cell carcinoma based on CT imaging: a multicentre, retrospective, diagnostic study. EClinicalMedicine 67:102385 10.1016/j.eclinm.2023.102385 PMC10796944 38261897

[25] Choi JH, Choi JY, Woo SK, Moon JE, Lim CH, Park SB et al (2024) Prognostic value of radiomic analysis using pre- and post-treatment (18)F-FDG-PET/CT in patients with laryngeal cancer and hypopharyngeal cancer. J Pers Med. 10.3390/jpm14010071 PMC10817325 38248772

[26] Cozzi D, Bicci E, Cavigli E, Danti G, Bettarini S, Tortoli P et al (2022) Radiomics in pulmonary neuroendocrine tumours (NETs). Radiol Med 127(6):609–615 10.1007/s11547-022-01494-5 PMC9130162 35538389

[27] Litvin AA, Burkin DA, Kropinov AA, Paramzin FN (2021) Radiomics and digital image texture analysis in oncology (review). Sovrem Tekhnologii Med 13(2):97–104 10.17691/stm2021.13.2.11 PMC8353717 34513082

[28] Nardi C, Tomei M, Pietragalla M, Calistri L, Landini N, Bonomo P et al (2021) Texture analysis in the characterization of parotid salivary gland lesions: a study on MR diffusion weighted imaging. Eur J Radiol 136:109529 10.1016/j.ejrad.2021.109529 33453571

[29] Mungai F, Verrone GB, Bonasera L, Bicci E, Pietragalla M, Nardi C et al (2021) Imaging biomarkers in the diagnosis of salivary gland tumors: the value of lesion/parenchyma ratio of perfusion-MR pharmacokinetic parameters. Radiol Med 126(10):1345–1355 10.1007/s11547-021-01376-2 34181206

[30] Chen M, Jiang Y, Zhou X, Wu D, Xie Q (2024) Dual-energy computed tomography in detecting and predicting lymph node metastasis in malignant tumor patients: a comprehensive review. Diagnostics (Basel). 10.3390/diagnostics14040377 PMC10888055 38396416

[31] Zhang W, Liu J, Jin W, Li R, Xie X, Zhao W et al (2024) Radiomics from dual-energy CT-derived iodine maps predict lymph node metastasis in head and neck squamous cell carcinoma. Radiol Med 129(2):252–267 10.1007/s11547-023-01750-2 38015363

[32] Berger T, Noble DJ, Shelley LEA, McMullan T, Bates A, Thomas S et al (2022) Predicting radiotherapy-induced xerostomia in head and neck cancer patients using day-to-day kinetics of radiomics features. Phys Imaging Radiat Oncol 24:95–101 10.1016/j.phro.2022.10.004 PMC9647222 36386445

[33] Xu XQ, Zhou Y, Su GY, Tao XW, Ge YQ, Si Y et al (2022) Iodine maps from dual-energy CT to predict extrathyroidal extension and recurrence in papillary thyroid cancer based on a radiomics approach. AJNR Am J Neuroradiol 43(5):748–755 10.3174/ajnr.A7484 PMC9089265 35422420

[34] Becker M (2000) Neoplastic invasion of laryngeal cartilage: radiologic diagnosis and therapeutic implications. Eur J Radiol 33(3):216–229 10.1016/s0720-048x(99)00144-8 10699738

[35] Pietragalla M, Nardi C, Bonasera L, Mungai F, Verrone GB, Calistri L et al (2020) Current role of computed tomography imaging in the evaluation of cartilage invasion by laryngeal carcinoma. Radiol Med 125(12):1301–1310 10.1007/s11547-020-01213-y 32415474

[36] Pecorari G, Lorenzi A, Caria M, Motatto GM, Riva G (2025) Clinical and pathological staging discrepancies in laryngeal cancer: a systematic review. Cancers (Basel). 10.3390/cancers17030455 PMC11815896 39941822

[37] Locatello LG, Bruno C, Gallo O (2021) Early glottic cancer recurrence: a critical review on its current management. Crit Rev Oncol Hematol 160:103298 10.1016/j.critrevonc.2021.103298 33716199

[38] Locatello LG, Pietragalla M, Taverna C, Bonasera L, Massi D, Mannelli G (2019) A critical reappraisal of primary and recurrent advanced laryngeal cancer staging. Ann Otol Rhinol Laryngol 128(1):36–43 10.1177/0003489418806915 30360641

[39] Mayerhoefer ME, Materka A, Langs G, Haggstrom I, Szczypinski P, Gibbs P et al (2020) Introduction to radiomics. J Nucl Med 61(4):488–495 10.2967/jnumed.118.222893 PMC9374044 32060219

[40] Diebold M, Becker L, Demerath T, Reisert M, Erny D, Braun A et al (2025) Non-invasive prediction of meningioma tumor grade by quantification of shape-based radiomics features and surface regularity. Clin Neuroradiol. 10.1007/s00062-025-01560-1 PMC13009051 40960507

[41] van Timmeren JE, Cester D, Tanadini-Lang S, Alkadhi H, Baessler B (2020) Radiomics in medical imaging-"how-to" guide and critical reflection. Insights Imaging 11(1):91 10.1186/s13244-020-00887-2 PMC7423816 32785796

[42] Guo R, Guo J, Zhang L, Qu X, Dai S, Peng R et al (2020) CT-based radiomics features in the prediction of thyroid cartilage invasion from laryngeal and hypopharyngeal squamous cell carcinoma. Cancer Imaging 20(1):81 10.1186/s40644-020-00359-2 PMC7661189 33176885

[43] Gardin I, Gregoire V, Gibon D, Kirisli H, Pasquier D, Thariat J et al (2019) Radiomics: principles and radiotherapy applications. Crit Rev Oncol Hematol 138:44–50 10.1016/j.critrevonc.2019.03.015 31092384

[44] Oki A, Nakamura S, Sakamoto J, Watanabe H, Miura M (2025) Can MRI radiomics predict neck metastasis at initial diagnosis in patients with squamous cell carcinoma of the tongue? Oral Radiol 41(3):388–395 10.1007/s11282-025-00810-9 39964626

[45] Struckmeier AK, Eichhorn P, Agaimy A, Buchbender M, Moest T, Lutz R et al (2020) Comparison of the 7th and revised 8th UICC editions (2020) for oral squamous cell carcinoma: how does the reclassification impact staging and survival? Virchows Arch 484(6):901–913 10.1007/s00428-023-03727-y PMC11186894 38191928

[46] Mes SW, van Velden FHP, Peltenburg B, Peeters CFW, Te Beest DE, van de Wiel MA et al (2020) Outcome prediction of head and neck squamous cell carcinoma by MRI radiomic signatures. Eur Radiol 30(11):6311–6321 10.1007/s00330-020-06962-y PMC7554007 32500196

[47] Bicci E, Nardi C, Calamandrei L, Pietragalla M, Cavigli E, Mungai F et al (2022) Role of texture analysis in oropharyngeal carcinoma: a systematic review of the literature. Cancers (Basel). 10.3390/cancers14102445 PMC9139172 35626048

[48] Kocak B, Durmaz ES, Ates E, Kilickesmez O (2019) Radiomics with artificial intelligence: a practical guide for beginners. Diagn Interv Radiol 25(6):485–495 10.5152/dir.2019.19321 PMC6837295 31650960

[49] Dogan MB, Gunduz N, Kazan HO, Cakici MC, Yildirim A, Erdem G (2025) Contribution of lesion shape features to the VI-RADS for predicting muscle invasion in bladder tumors. Eur J Radiol 187:112104 10.1016/j.ejrad.2025.112104 40215707

[50] Zhang L, Du Q, Shen M, He X, Zhang D, Huang X (2025) Interpretable model based on MRI radiomics to predict the expression of Ki-67 in breast cancer. Sci Rep 15(1):13318 10.1038/s41598-025-97247-1 PMC12006291 40246899

[51] Becker M, de Vito C, Dulguerov N, Zaidi H (2023) PET/MR imaging in head and neck cancer. Magn Reson Imaging Clin N Am 31(4):539–564 10.1016/j.mric.2023.08.001 37741640

[52] Safakish A, Sannachi L, Moslemi A, Pejović-Milić A, Czarnota GJ (2024) Deep texture analysis—enhancing CT radiomics features for prediction of head and neck cancer treatment outcomes: a machine learning approach. Radiation (Tokyo) 4(1):50–68

